# A hospital qPCR-based survey of 10 gastrointestinal parasites in routine diagnostic screening, Marseille, France

**DOI:** 10.1017/S0950268819000165

**Published:** 2019-02-22

**Authors:** E. Menu, C. Mary, I. Toga, D. Raoult, S. Ranque, F. Bittar

**Affiliations:** 1 Aix Marseille University, IRD, APHM, MEPHI, IHU-Méditerranée Infection, Marseille, France; 2 Aix Marseille University, IRD, APHM, VITROME, IHU-Méditerranée Infection, Marseille, France

**Keywords:** Enteric parasites, France, microsporidia, protozoa, qPCR

## Abstract

There is a scarcity of recent epidemiological data on intestinal parasitic infections in France. We conducted a prospective study aimed at estimating the prevalence of 10 enteric parasites in Marseille, France, using real-time polymerase chain reaction (PCR)-based diagnosis. A total of 643 faeces from 488 patients referred to the Parasitology-Mycology Laboratory of the University Hospital of Marseille over a 6 months period were included. DNA was extracted using a semi-automated method. Parasites of interest were detected using singleplex quantitative PCRs (qPCRs). For positive samples, the *Blastocystis* subtype was determined by sequence analysis. During the study, the overall prevalence of enteric parasites was 17%. *Blastocystis sp.* was the most frequent species (10.5%), followed by *Dientamoeba fragilis* (2.3%) and *Giardia intestinalis* (2.3%). The prevalence of other parasites was <1% each. The ST3 *Blastocystis* subtype was predominant (43.6%) and the other subtypes identified were ST1, ST2, ST4 and ST6. This is the first time that a qPCR-based diagnosis has been used to survey the prevalence of 10 enteric parasites in a French University Hospital. This study confirms that fast, specific, sensitive and simultaneous detection in a single stool sample by qPCR clearly outperforms conventional microscopy-based diagnosis. Furthermore, qPCR is particularly well suited to surveying gastroenteritis agents.

## Introduction

Intestinal parasitic disease remains one of the greatest health problems in developing countries. The World Health Organization estimates that around 3.5 billion people worldwide are affected and that 450 million show symptoms of an illness [1]. Much attention has been paid to enteric parasites in developing countries. In contrast, the current situation of enteric parasitic diseases in Europe is poorly understood. First, due to the lack of an operational surveillance system and under-reporting, confirmed cases of intestinal parasitic infections are often not reported. Thus, available data are fragmented and the prevalence of such diseases might be underestimated. Second, the frequency of intestinal parasitic diseases might also be subjected to changes in industrialised countries as a consequence of climate change, globalisation, increasing frequency of travels and worldwide international exchanges, including immigration and the adoption of children from endemic regions [2]. Therefore, it appears crucial to conduct epidemiological studies in industrialised countries. Regarding protozoa parasites, *Cryptosporidium parvum*/*hominis*, *Blastocystis sp.*, *Entamoeba* spp. as well as *Giardia intestinalis* and *Dientamoeba fragilis* are the most common species associated with human diarrhoea worldwide [3], although the pathogenic potential of *Blastocystis* sp. and *D. fragilis* remains controversial. In fact, recent publications have reported *D. fragilis* as a commensal in children [4,5]. These species, along with others such as *Cyclospora cayetanensis*, *Balantidium coli* and *Cystoisospora belli* and two microsporidia, *Enterocytozoon bieneusi* and *Encephalitozoon intestinalis*, should therefore be considered in such surveillance studies.

While microscopic examination of stool samples remains the gold standard for the diagnosis of parasitic infections, it is time-consuming, laborious and requires substantial technical expertise. In contrast, rapid diagnostic methods such as polymerase chain reaction (PCR)-based assays have been developed to improve sensitivity and specificity of the detection of enteric parasites, including helminths, protozoa and microsporidia [6, 7]. While most of the published studies focus on a limited number of intestinal protozoa [8, 9], new PCR-based diagnostic tools can detect a large range of intestinal parasites in a single human stool sample and are particularly suitable for epidemiological surveys [10, 11]. Their high sensitivity allows the detection of low parasite levels and the use of quantitative assays, enabling the parasitic load to be quantified, which might be useful for the post-treatment monitoring of the patients [12–14]. Furthermore, detecting multiple intestinal parasites using a standardised protocol enhances the reproducibility of results.

This work is the first prospective, monocentric, French epidemiological study aiming at estimating the occurrence of eight protozoans: *Blastocystis sp.*, *C. parvum/hominis*, *C. cayetanensis*, *D. fragilis*, *G. intestinalis*, *B. coli*, *Entamoeba histolytica* and *C. belli* and two microsporidia: *E. bieneusi* and *E. intestinalis*, using singleplex real-time quantitative PCR (qPCR) analysis. Microscopy and qPCR results were also compared.

## Materials and methods

### Samples collection, ethical norms and routine microscopic examination

Faecal samples were collected at the Parasitology-Mycology Laboratory at La Timone University Hospital in Marseille during routine parasitological examinations of stool samples from patients between January 2017 and July 2017. Intestinal parasites in stool specimens were assessed during routine medical visits. Patients received written laboratory work-up reports. qPCRs were performed on surplus stool samples. Patient's characteristics were obtained from a retrospective, non-interventional review of medical charts and laboratory results. According to French law, the patients were informed that their samples and clinical data may be used for research purposes and retained the right to oppose to the use of their anonymous medical data for such purposes. Therefore, neither dedicated ethical approval nor individual patient consent was required for this type of study (Loi no 2012–300 du 5 mars 2012 and Décret no 2016–1537 du 16 novembre 2016 published in the ‘*Journal Officiel de la République Française*’). For each patient, the reason for the consultation and sampling were retrieved from the hospital management system database and were classified into five groups: gastro-intestinal symptoms including abdominal pain, diarrhoea, rectal bleeding, abdominal meteorism (bloating) and constipation (some of these symptoms were found concurrently); recent travel to endemic areas; hyper-eosinophilia; immunosuppression (HIV, transplant recipient, chemotherapy); and undetermined reason. Of note that some patients might belong to several groups. Upon arrival, microscopic examination of each stool sample was routinely performed by an experienced operator, using direct saline solution, iodine mounts and formol-ethyl acetate concentration techniques. *Cryptosporidium* spp. and Microsporidia have exclusively been sought at the request of the prescriber. In these cases, a modified Ziehl–Neelsen stain and in-house PCR were performed, respectively.

### DNA extraction from stool samples

DNA extraction was performed daily using the EZ1 (Qiagen GmbH, Hilden, Germany) automated protocol with mechanical, chemical and enzymatic pre-treatment [15]. The extraction protocol was adapted for stool processing as follows: 200 mg of stool sample was added to 350 µl of G2 lysis buffer (Qiagen GmbH) in a tube containing glass powder (acid-washed glass beads 425–600 µm, Sigma-Aldrich, Saint Quentin Fallavier, France) and then disrupted in a FastPrep-24 grinder (MP Biomedicals, Illkirch-Graffenstaden, France) at maximum power for 40 s. After 10 min of incubation at 100 °C to allow complete lysis, tubes were centrifuged at 10 000 ***g*** for 1 min. A 200 µl of supernatant was then added to a tube containing 20 µl of Proteinase K, and incubated overnight at 56 °C. Finally, the automated protocol using the EZ1 Advanced XL extractor was performed as described by the manufacturer. Extracted DNA was eluted in 200 µl.

To control for both DNA extraction quality and the absence of PCR inhibitors, a eubacterial 16S rRNA qPCR was performed on each DNA, as previously described [16]. Extraction was repeated in the event of a negative result which indicates the presence of PCR inhibitors in the sample.

### Singleplex qPCR amplification and detection

Ten different specific primer pairs and Taqman™ (Eurogentec^®^, Seraing, Belgium) hydrolysis probes targeting the following species: *Blastocystis* sp., *C. parvum/hominis*, *C. cayetanensis*, *C. belli*, *D. fragilis*, *E. bieneusi*, *E. intestinalis*, *B. coli*, *E. histolytica* and *G. intestinalis* were used in singleplex assays (Table 1). All primer sequences and PCR conditions have been previously described [17–26]. Briefly, each qPCR reaction was conducted in a 20 µl total volume containing 10 µl of Master mix (Roche Diagnostics GmbH, Mannheim, Germany), 0.5 µl of each primer, 0.5 µl of probe, 3 µl of distilled water, 0.5 µL of UDG and 5 µl of DNA. Analyses were performed using a CFX96™ Real-Time PCR detection system (BIO-RAD, Life Science, Marnes-la-Coquette, France). Amplification reaction was performed directly after DNA extraction as follows: 2 min of incubation at 50 °C, 5 min of incubation at 95 °C, followed by 40 cycles of 5 s at 95 °C and 30 s at 60 °C. The qPCR results were considered negative when the Ct value exceeded 37 or when no amplification was obtained, as described in previous studies [11, 17].

**Table 1.** List of primers and probes used in this study

Organism	Name	Primers/probes	Target region	References
*Balantidium coli*	BcoliF	5′-TGCAATGTGAATTGCAGAACC-3′	ITS-1	[17]
	BcoliR	5′-TGGTTACGCACACTGAAACAA-3′		
	BcoliP	5′-FAM-CTGGTTTAGCCAGTGCCAGTTGC-TAMRA-3′		
*Blastocystis* sp.	Blasto FWD F5	5′-GGTCCGGTGAACACTTTGGATTT-3′	18S	[18]
	Blasto R F2	5′-CCTACGGAAACCTTGTTACGACTTCA-3′		
	Blasto probe	5′-FAM-TCGTGTAAATCTTACCATTTAGAGGA-MGBNFQ-3′		
*Cryptosporidium parvum/hominis*	1PS_F	5′-AACTTTAGCTCCAGTTGAGAAAGTACTC-3′	Hsp70 gene	[19]
	1PS_R	5′-CATGGCTCTTTACCGTTAAAGAATTCC-3′		
	Crypt_P	5′-FAM-AATACGTGTAGAACCACCAACCAATACAACATC-TAMRA-3′		
*Cyclospora cayetanensis*	Cyclo250F	5′-TAGTAACCGAACGGATCGCATT-3′	18S	[20]
	Cyclo350R	5′-AATGCCACGTAGGCCAATA-3′		
	Cyclo281T	5′-FAM-CCGGCGATAGATCATTCAAGTTTCTGACC-TAMRA-3′		
*Cystoisospora belli*	Ib-40F	5′-ATATTCCCTGCAGCATGTCTGTTT-3′	ITS2	[21]
	Ib-129R	5′-CCACACGCGTATTCCAGAGA-3′		
	Ib-81Taq	5′-FAM-CAAGTTCTGCTCACGCGCTTCTGG-TAMRA-3′		
*Dientamoeba fragilis*	Df-124F	5′-CAACGGATGTCTTGGCTCTTTA-3′	18S	[22]
	Df-221R	5′-TGCATTCAAAGATCGAACTTATCAC-3′		
	Df-172revT	5′-FAM-CAATTCTAGCCGCTTAT-BHQ1-3′		
*Encephalitozoon intestinalis*	FEI1	5′-GCAAGGGAGGAATGGAACAGAACAG-3′	18s	[23]
	REI1	5′-CACGTTCAGAAGCCCATTACACAGC-3′		
	PEI1	5′-FAM-CGGGCGGCACGCGCACTACGATA-TAMRA-3′		
*Entamoeba histolytica*	Ehf	5′-AACAGTAATAGTTTCTTTGGTTAGTAAAA-3′	18s	[24]
	Ehr	5′-CTTAGAATGTCATTTCTCAATTCAT-3′		
	Ehp	5′-FAM-ATTAGTACAAAATGGCCAATTCATTCA-TAMRA-3′		
*Enterocytozoon bieneusi*	FEB1	5′-CGCTGTAGTTCCTGCAGTAAACTATGCC-3′	18S	[25]
	REB1	5′-CTTGCGAGCGTACTATCCCCAGAG-3′		
	PEB1	5′-FAM-ACGTGGGCGGGAGAAATCTTTAGTGTTCGGG-TAMRA-3′		
*Giardia intestinalis*	Giardia-80F	5′-GACGGCTCAGGACAACGGTT-3′	18S	[26]
	Giardia-127R	5′-TTGCCAGCGGTGTCCG-3′		
	Giardia-105T	5′-FAM-CCCGCGGCGGTCCCTGCTAG-BHQ1-3′		

### Standard curves using plasmid templates for quantification

Serial dilutions of plasmids designed with target nucleotide sequences were used to establish standard curves using a dilution range of 10^2^ to 10^7^ copies of plasmid DNA in the qPCR assay. Plasmid DNA were synthetised by Eurogentec^®^; the targeted gene was cloned in pUC57 by EcoRV. Lyophilised plasmids were rehydrated to one target-sequence copy per plasmid. A set of plasmid dilutions was included in each qPCR assay to estimate the number of target copies and to monitor analytical sensitivity. Two dilutions (10^3^ and 10^4^ copies) of each plasmid were included in each assay as positive controls.

### Amplification of the SSU rDNA gene and *Blastocystis* sp. molecular subtyping

DNA sequencing was used for *Blastocystis sp.* subtyping. Each *Blastocystis sp.*-positive DNA sample was subjected to a standard PCR assay using the *Blastocystis sp.*-specific primers BL18SPPF1 (5′-AGTAGTCATACGCTCGTCTCAAA-3′) and BL18SR2PP (5′-TCTTCGTTACCCGTTACTGC-3′), designed by Poirier *et al*. [27]. These primers target a 320–342 bp DNA fragment in the *Blastocystis sp.* SSU rRNA gene, the nucleotide sequence of which varies depending on the subtype (ST). Amplification was performed in a 50 µl total volume with the AmpliTaq Gold^®^ 360 protocol (Thermo Fisher Scientific, France). Following denaturation at 95 °C for 15 min, 40 cycles of amplification were performed with a 2720 Thermal Cycler™ (Applied Biosystems, Courtaboeuf, France) as follows: 30 s at 95 °C, 30 s at 59 °C and 30 s at 72 °C. The amplification products were assessed by electrophoresis using 1.5% agarose gel and SYBR™ Safe DNA Gel Stain (Invitrogen ™, Carlsbad, USA). PCR products were purified using MultiScreen^®^ PCR (Meck Millipore, Darmstadt, Germany) and the sequencing reaction was carried out using a DNA sequencing kit (BigDye Terminator Cycle Sequencing v1.1 Ready Reactions; AB Applied Biosystems) according to the manufacturer's instructions. Sequencing products were purified, and electrophoresis was performed with a 3130 Genetic Analyzer (Applied Biosystems). The nucleotide sequences were assembled and corrected using the CodonCode Aligner (Centerville, MA, USA) software and compared with those available in the GenBank database, using the BLASTn (http://www.ncbi.nlm.nih.gov/BLAST) software.

### Data analysis

For qualitative variables, the *χ*^2^ (when applicable) or the Fisher's exact test was used. For quantitative variables, normal distribution was assessed using the Kolmogorov–Smirnov test and the Student's *t* test or the Mann–Whitney test were used when applicable. Statistical analyses were performed using the GraphPad Prism, version 6.0 (La Jolla, CA) software.

### Nucleotide sequence accession numbers

All sequences obtained in this work were deposited in GenBank database under accession numbers MG865904 to MG865961.

## Results

### Prevalence and diseases

This study includes a total of 643 stool samples (from 488 patients; 111 patients of them provided more than one sample) that were submitted to the Parasitology Department of La Timone Hospital (3400 beds; >1 000 000 consultations/year) in Marseille for routine microscopic examination between January 2017 and July 2017. Patients’ ages ranged from 1 to 97 years and the sex ratio was 1.09. No PCR inhibitors were detected by eubacterial 16S rRNA qPCR; the average Ct for all samples was 18.33 ± 4.06. Among these patients, 83 (17%, 95% confidence interval (95% CI) 13.8–20.7) were found to be positive for at least one of the 10 enteric parasites tested (Table 2). *Blastocystis sp.* was the most common, with a 10.5% prevalence (51 patients) (95% CI 7.9–13.6), followed by *G. intestinalis* and *D. fragilis* with 2.3% prevalence (11 patients) (95% CI 1.2–4) each. The prevalence of the remaining investigated parasites was as follows: three patients (0.6%, 95% CI 0.1–1.9) for *C. cayetanensis*, two patients (0.4%, 95% CI 0–1.6) for each *C. belli*, *E. bieneusi* and *C. parvum/hominis* and one patient (0.2%, 95% CI 0–1) for *E. intestinalis.* Neither *E. histolytica* nor *B. coli* were detected in the tested samples. Of the 488 patients, 283 (58%, 95% CI 53.5–62.4) had gastro-intestinal symptoms, 80 (16.4%, 95% CI 13–20) had travelled to tropical or developing countries during the last 6 months, 26 (5.3%, 95% CI 3.6–7.8) consulted because of hyper-eosinophilia and 169 (34.6%, 95% CI 30.4–39) were immunosuppressed, and in 40 of cases (8.20%, 95% CI 6–11) the reasons for performing the examination were not provided. For the remaining 69 patients, the reasons for stool examination did not fit any of the aforementioned groups and include unexplained itching and allergies. Among positive qPCR patients, six of them had HIV (one patient with *Cryptosporidium* spp., two patients with *C. cayetanensis*, two patients with *Cytoisospora belli* and one patient with *E. bieneusi*) and two of them had renal transplant (one patient with *Cryptosporidium* spp. and one patient with *E. bieneusi*). Among the 11 positively diagnosed patients with *G. intestinalis*, nine of them were associated with diarrhoea. Finally, no significant association was found linking the detected enteric parasites with the age range of patients, neither with the time of sample collection nor the reason for consultation and sampling.

**Table 2.** Intestinal parasites in stool samples from the department of parasitology at La Timone hospital, Marseille (*n* = 643) as detected with microscopy and qPCR

	Positive samples by qPCR (%) (*n* = 643 stool samples)	Positive patients by qPCR (%) (*n* = 488 patients)	Positive samples by microscopy
*Balantidium coli*	0 (0)	0 (0)	0
*Blastocystis spp.*	62 (9.6)	51 (10.5)	2
*Cryptosporidium spp.*	2 (0.3)	2 (0.4)	0
*Cyclospora cayetanensis*	3 (0.5)	3 (0.6)	0
*Cystoisospora belli*	2 (0.3)	2 (0.4)	0
*Dientamoeba fragilis*	11 (1.7)	11 (2.3)	0
*Encephalitozoon intestinalis*	1 (0.2)	1 (0.2)	0
*Entamoeba histolytica*	0 (0)	0 (0)	0
*Enterocytozoon bieneusi*	5 (0.8)	2 (0.4)	0
*Giardia intestinalis*	13 (2)	11 (2.3)	5
Total	99	83	7

### Comparison between qPCR and microscopy

Overall, qPCR and microscopic examination were positive in 99 (15.4%, 95% CI 12.7–18.5) and seven (1.1%, 95% CI 0.5–2) of the 643 samples, respectively (*P* < 0.0001). While the detection of *G. intestinalis* was positive in five samples using both methods, eight additional samples were detected positive for *G*. *intestinalis* via qPCR (Table 2). *Cyclospora cayetanensis*, *C. belli*, *C. parvum/hominis* and *D. fragilis* were only detected by qPCR. Of note that low levels of *Blastocystis* sp. have not been notified in routine microscopic examination because they stand within the normal, ‘non-pathogenic’ range (i.e. ⩽5 cysts per high-power field). Moreover, *Cryptosporidium* spp. and *Microsporidia* spp. were exclusively searched at the request of the prescriber/doctor. Thus, the overall detection rates, calculated excluding *Blastocystis sp.*, *Cryptosporidium spp.* and *Microsporidia*, were 4.5% (*n* = 29) (95% CI 3–6.5) and 0.8% (*n* = 5) (95% CI 0.3–1.9) for qPCR and microscopy (*P* < 0.0001), respectively.

### Poly-parasitism

In 15 samples, two or three distinct species were concomitantly detected. *Blastocystis* sp. and *D. fragilis* were found in eight patients; *Blastocystis* sp. and *G. intestinalis* in three patients; and *Blastocystis* sp. and *C. parvum/hominis*, *Blastocystis* sp. and *E. intestinalis*, and *C. cayetanensis* and *C. belli* in one patient each. In one patient, we detected *Blastocystis* sp., *C. cayetanensis* and *C. belli*.

### qPCR quantification

**Fig. 1.**
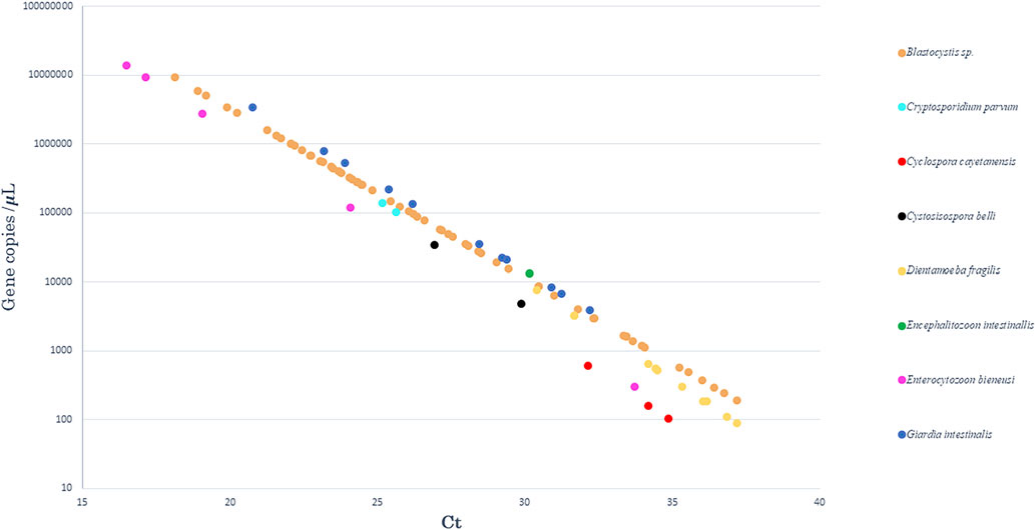
Results obtained after quantification by qPCR. The curve representing the number of gene copies, as a function of qPCR Ct values, in positive samples for each of the eight enteric parasites tested.

Standard curves were analysed to determine the efficiency of qPCR reactions. The standards had a linear quantification range from 1 × 10^2^ to 1 × 10^7^gene copies per 5 µl of plasmid DNA, and the limit of detection was 10 copies per reaction (data not shown). The efficiency, slope of the standards, correlation coefficient (**r**^2^) and intercept ranged from 80.5% to 94.7%, −3.898 to −3.455, 0.970 to 0.999 and 41.915 to 46.239, respectively. The calculated gene copy number in positive samples is plotted for each intestinal pathogen in Figure 1.

### Blastocystis subtyping

**Fig. 2.**
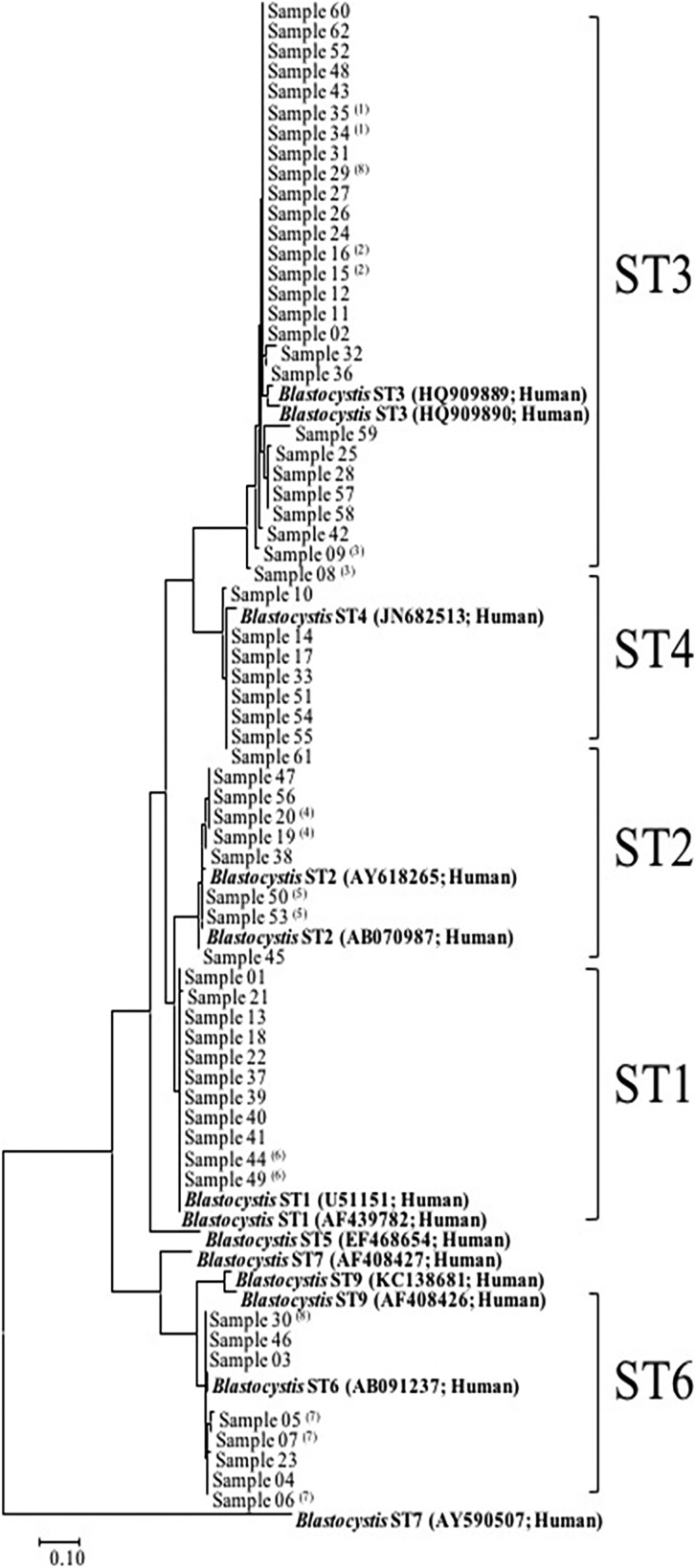
Phylogenetic relationship between the SSD rDNA sequence of *Blastocystis*. The molecular phylogenetic analysis was carried out using the Maximum Likelihood method based on the Tamura–Nei model. ST, subtype; (*n*): patient number in case of multiple samples.

Following the sequence analyses of the 62 *Blastocystis*-positive samples (51 patients), five STs were detected (Fig. 2). ST3 was the most frequent (*n* = 27, 43.6%, 95% CI 31–56.7), followed by ST1 (*n* = 11, 17.7%, 95% CI 9.6–29.9), ST2 (*n* = 8, 12.9%, 95% CI 6–24.4), ST4 (*n* = 8, 12.9%, 95% CI 6–24.4) and ST6 (*n* = 8, 12.9%, 95% CI 6–24.4). In cases where a given patient provided several samples, the same subtype was found in all related samples except for one patient (patient 8), in whom we found ST3 and ST6 in samples 29 and 30, respectively. A phylogenetic tree was constructed to illustrate the relationship between the different subtypes found in our samples (Fig. 2). There was no significant correlation between patient demographics, disease categories or co-detection with another parasite and the *Blastocystis* subtype.

## Discussion

This work is the first prospective epidemiological survey of 10 enteric parasites based on qPCR conducted in France. It highlights the enhanced specificity and sensitivity of qPCR compared with microscopy for the diagnosis of these enteric parasites. Despite relatively high costs, a growing number of clinical laboratories are equipped with automated extraction systems and qPCR thermal cyclers. Increased diagnostic performance is particularly important for enhancing patients’ care but also for detecting outbreaks. When investigating an outbreak, enhanced diagnosis and case detection makes it easier to trace the source and improve the control of the outbreak [28]. We will successively discuss our findings for each pathogen.

*Blastocystis* sp. was found in 10.5% (51/488) of the patients, making it the most common enteric parasites in our study. This is not surprising, since *Blastocystis* sp. are among the most frequently observed intestinal parasites in humans with a prevalence ranging between 0.5% and 60% across the world [29]. The prevalence found in our survey throughout winter and spring is closely similar to the prevalence (13.7%) that was reported in the winter in a recent multi-centre study in France [30]. However, it remains lower than the prevalence (35.2%) found in a recent Spanish study [31]. The pathogenicity of *Blastocystis* sp. is still controversial [32]. While, in our study, we did not find any significant correlation between *Blastocystis* sp. detection and the reason for the consultation, abdominal pain was significantly more frequently reported in *Blastocystis* sp. carriers. The prevalence of *Blastocystis* in the population has been confirmed by both qPCR and standard PCR. The low sensitivity of microscopy can be explained by the fact that the *Blastocystis* is notified only when high levels (i.e. >5 cysts per high-power field) are present in stool samples. The subtype distribution in the study population showed five different subtypes: ST1, ST2, ST3, ST4 and ST6. Subtypes 1–4 have been pointed out as the most common subtypes in humans [30, 33]. In line with our findings, a previous study identified ST3 as the predominant subtype [33]. Nevertheless, another recent work performed in northern Spain detected ST2 more frequently than the other subtypes together [31]. ST1 was relatively frequent in our study group (17.7%) compared with other European studies (Swedish patients [34] and Spanish individuals [31]), but is consistent with one previous French study [35]. In addition, similar results for the remaining subtypes have also been reported in Sweden [34]. The relative occurrence of subtypes 1–4 and 6 suggests that heterogeneity in transmission efficiency to humans may exist between them. The ST4 is mainly found in Europe [34] and has been associated with infectious diarrhoea [31], but not in our work. The relatively high prevalence of ST6 observed in this study, which has mainly been described in birds so far, may suggest a zoonotic transmission [30, 36].

Concerning *G. intestinalis*, which is a protozoan often responsible for diarrhoea, it was detected using qPCR with a prevalence of 2.3%, and is the second most prevalent in our study. This is in agreement with previous studies that estimated that the prevalence of *G. intestinalis* ranges between 2% and 7% in industrialised countries [3]. We also noticed that giardiasis remains underdetected by microscopy. Indeed, the quality of the microscopic investigation remains technician-dependent and time-consuming. In developed countries, where a limited number of positive samples have been examined and where the parasite levels are typically low in positive samples, technicians’ performance is likely to be suboptimal. In our work, the samples missed by microscopy revealed significantly higher Ct values (*P* = 0.0034), indicative of a lower number of parasites, which has also been reported elsewhere [37]. Except for *G. intestinalis*, for which 81.8% (9/11) of the infected patients presented with diarrhoea, the other detected parasites were not associated neither with a symptom nor with a group of patients.

*Dientamoeba fragilis* is a flagellate protozoan parasite of the human gastro-intestinal tract, the biological cycle of which remains partly undetermined [38]. The prevalence of dientamoebiasis equals that found for giardiasis in this study, with a prevalence of 2.3%. Previous surveys showed that the prevalence varies from 0.4% (in patients with gastrointestinal discomfort) to 82.9% (in children infected with other gastrointestinal protozoa) [38]. Although recent studies have reported *D. fragilis* as a commensal in children [4, 5], the detection of this parasite was neither statistically associated with diarrhoea nor showed a bias towards children. A study conducted in the Netherland [39] revealed a rate of co-infection with *Blastocystis* sp. in a half of the cases of dientamoebiasis in paediatric patients. Similarly, we documented, in our survey, a high rate of combination with *Blastocystis* sp. (72.7%, 8 patients/11). It is notable that there was no significant correlation between *D. fragilis* infection and *Blastocystis* subtypes.

On explanation for the low number of positive stool samples for *Cryptosporidium* spp. (0.4%, two patients) might be that our study was carried out from January to July 2017, while in developed countries, a peak in the prevalence of cryptosporidiosis is observed during summer months [40]. In addition, both patients were receiving immunosuppressive therapy; one was a renal transplant recipient and the other was a patient co-infected by HIV and HCV who presented with diarrhoea after returning from India, a highly endemic area [41].

Microsporidiosis, an opportunistic infection caused by obligate intracellular microsporidia fungi species, has been particularly well documented in immunocompromised patients [42]. In this study, we found four *E. bieneusi*-positive samples in an HIV-infected patient with a low (<100/mm^3^) CD4 count at the time of collection. Another patient was a renal transplant recipient. *Encephalitozoon intestinalis* was also found in a patient suffering from diarrhoea without a fever, who had lived in Cambodia and died before being tested for HIV.

The two *C. cayetanensis*-positive patients were immigrants from Albania and HIV-positive. Both were co-infected with *C. belli*. Due to the lack of microscopic detection of these two parasites in both patients, we cannot exclude a cross-reactivity between *C. cayetanensis* and *C. belli* PCRs. One of these two patients was also infected with *Blastocystis sp.* Cases of cyclosporiasis have been described previously in Germany [43].

Finally, we did not detect any patients with *E. histolytica* or *B. coli* during our study. In fact, in developed countries, *E. histolytica* infections are rare and often related to travel to endemic regions [44] although sporadic and epidemic cases of *B. coli* have been described in Europe [45].

## Conclusion

In this prospective hospital-based epidemiological survey, we report the occurrence of 10 enteric parasites, including eight protozoans and two microsporidia species, in Marseille, France. It highlights a relatively high prevalence of *G. intestinalis* and *D. fragilis*, second only to *Blastocystis* sp. The detection of these parasites, mainly by qPCRs, provides further evidence of qPCR superiority and questions the current use of microscopy as the diagnostic gold standard. Moreover, our results emphasise the value of qPCR-based assays in stools for surveying multiple infectious gastroenteritis agents. Similar studies have been previously performed in other European countries. However, this work is the first study to use qPCR to detect gastrointestinal parasites in routine diagnostic screening in France, which can help to understand the current situation of enteric parasitic diseases in Europe. Further epidemiological surveys should aim at identifying risk factors associated with these parasites, including seasonality or eco-geographical factors.
